# A Case of Abscess in the Groin Attended with Symptoms of Hernia, from Which Two Lumbrici Teretes and Afterwards Fæcal Matter Were Discharged, and the Patient Recovered

**Published:** 1845-09

**Authors:** Thomas Howell

**Affiliations:** Risborough


*A case of Abscess in the Groin attended with symptoms of Hernia, from which two lumbrici teretes and afterwards fæcal matter were discharged, and the patient recovered.* By Thomas Howell, Esq., of Risborough. (Communicated by Alexander

Shaw, Esq.)—John S——, aged seventy, was attacked with symptoms of strangulated hernia. The author found a small hard tumor, about the size of a hen's egg, in the right inguinal region, lying over Poupart's ligament, about midway between the internal and external abdominal rings. At first he supposed it to be a hernia, but on careful examination, could detect no impulse on coughing, nor detect a neck towards the internal ring, and the tumour was not in the precise situation of inguinal hernia, and he was led to conclude that it was an enlarged inguinal gland. The symptoms were relieved by calomel and opium and a dose of castor oil, which acted freely. After two or three days, phlegmonous inflammation took place, and on fluctuation being perceived, the part was opened and four ounces of pus discharged. On the poultice being removed next morning, two worms, (lumbrici teretes,) each from five to six inches long, were found in it. After that, fæculent matter continued to escape from the wound for three weeks. He also passed, per anum, fæces, but not of so bright or yellow a color as that from the groin. The discharge from the wound gradually diminished, and in five weeks the author succeeded, by means of pressure, in healing the orifice. The patient stated that he never had any hernia.

Mr. Cæsar Hawkins remarked that there was one point in the paper which he could not allow to pass without observation, particularly as he believed that it had in some other cases been the source of error. He alluded to the absence of impulse in the tumor from coughing; a circumstance which Mr. Howell and his partner had regarded as indicative of the hernia not being strangulated. Now he (Mr. Hawkins) should have considered this one reason for deciding that the hernia was strangulated, for in nineteen cases out of twenty of this obstruction no impulse would be communicated to the hand on coughing.

Dr. Chowne had, some years since, seen a case bearing in some degree on that under consideration. The case was one of constipation, in which a considerable quantity of quicksilver was administered. A tumor formed in the groin, which eventually sloughed, and two ounces of quicksilver escaped with the fæces from the opening thus made. The case was attended with symptoms similar to those observed in Mr. Howell's case. The patient recovered.

Mr. Arnott mentioned a case which had come under his care some years since in the Middlesex Hospital. An abscess formed on the right side, in the same situation as that in Mr. Howell's case. Sloughing took place, and a discharge of fæ-

cal matter ensued. The patient, a young girl, eventually did well. With regard to the point of diagnosis of strangulated hernia, alluded to in the paper, and subsequently by Mr. Hawkins, he (Mr. Arnott) had an illustration of the accuracy of Mr. Hawkins's remark in the very last case of hernia in which he had operated. It was a case of strangulated femoral hernia in an old woman. There was no impetus given to the tumor by coughing. He explained to the pupils that the absence of this symptom was a reason for operating, as it was an evidence of the existence of strangulation. The operation was performed, and the patient did well. Abscesses, in connexion with the intestine, were often a source of great anxiety to the surgeon. He had lately been called to a case in which a gentleman thought that he had committed a mistake by opening what he did not suppose was a hernial tumor. He had concluded, from the absence of impulse on coughing in the tumor, that it was an abscess, and had plunged his knife into the swelling. Finding that this proceeding was followed by a discharge of a quantity of fæcal matter, he became alarmed. This abscess was situated on the left side, not, as was usual, on the right. There was no hernia, but a communication between the intestine and the abscess, no doubt, existed. The patient did well.—*London Lancet.*

